# Continuous Focusing of Particles by AC-Electroosmosis and Induced Dipole Interactions

**DOI:** 10.1021/acs.langmuir.4c02135

**Published:** 2024-09-13

**Authors:** Harm T. M. Wiegerinck, Jeffery A. Wood, Jan C. T. Eijkel, Rob G. H. Lammertink, Itzchak Frankel, Antonio Ramos

**Affiliations:** † Soft Matter, Fluidics and Interfaces, MESA+ Institute for Nanotechnology, University of Twente, 7500 AE Enschede, The Netherlands; ‡ BIOS/The Lab-on-a-Chip group, MESA+ Institute for Nanotechnology, University of Twente, P.O. Box 217, 7500 AE Enschede, The Netherlands; § Department of Aerospace Engineering, Technion - Israel Institute of Technology, Haifa 32000, Israel; ∥ Departamento de Electronica y Electromagnetismo, Universidad de Sevilla, Avenida Reina Mercedes, s/n, 41012 Sevilla, Spain

## Abstract

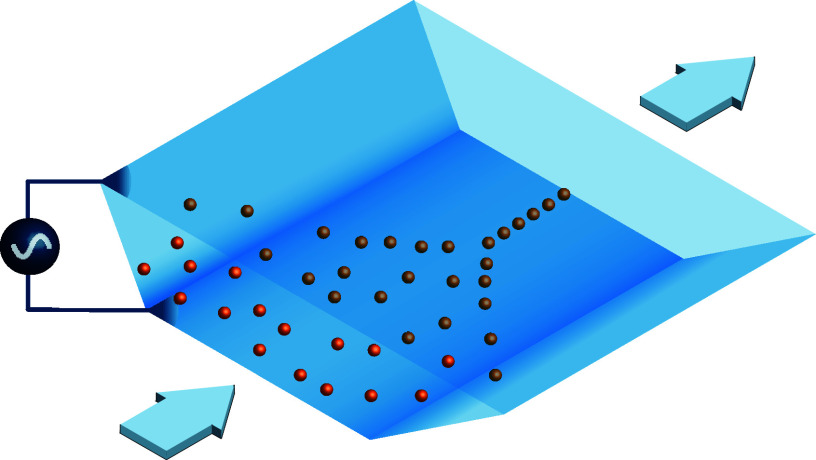

Continuous particle focusing by using microfluidics is an effective method for separating particles, cells, or droplets for analytical purposes. Previously, it was shown that an alternating current across rectangular microchannels with slightly deformed side walls results in vortex flow patterns caused by alternating current electroosmosis (AC-EOF) and could lead to particle focusing. In this work, we explore this mechanism by experimentally studying the particle focusing behavior for various fluid flow velocities through a microchannel. Since it is unlikely that the particles are kept in their focused position solely by convection, a theoretical force balance between the hydrodynamic and the induced dipole force was determined. In our experiments, it was found that there is no substantial effect of the pressure-driven fluid velocity on the particle focusing velocity within the studied range. From the theoretical force balance calculations, it was determined that while the addition of the induced dipole force can still not completely describe the experimentally observed particle focusing, the induced dipole can be strong enough to overcome the hydrodynamic force. Finally, it is hypothesized that under specific circumstances, including a repulsive electrostatic force between a particle and electrode wall can complete the theoretical particle focusing force balance. Alternative phenomena that could also play a role in particle focusing are proposed.

## Introduction

The continuous separation of micron-sized particles from aqueous dispersions is important for a wide range of analytical applications from the counting of bacteria in the food industry and discriminating different biological cell types to the analysis of the size distribution of oil droplets.^1,2^ There are broadly two different classes of particle separation techniques in microfluidics: passive and active techniques. Some passive techniques rely on flow effects, such as Dean vortices, inertial lift, or shear forces to concentrate particles, which are categorized as inertial particle focusing techniques in literature.^1−4^ Alternatively, membranes or filters could be used that separate the particles based on size, though this has the disadvantage that it could be clogged over time by the particle accumulation leading to the loss or damage of particles.^1,5^ This makes membrane techniques less suitable for analytical purposes. Active particle focusing methods rely on external applied force fields, such as an electric field,^6,7^ magnetic field,^8^ or concentration gradient.^9−11^ Furthermore, combinations of different forces^12^ or hybrid methods combining passive and active methods were studied in literature.^1,13^ While active particle focusing techniques usually consume more energy compared to passive particle focusing, this comes with the benefit of a higher particle recovery which is beneficial for analytical applications where a high sensitivity is required.^1^

Electric field-assisted particle focusing can be subdivided into methods that use direct or alternating current. The mechanisms that induce particle focusing in direct current fields include electrophoresis^14^ and electroosmosis.^15^ However, direct electric fields require electrode reactions in continuous particle focusing systems that could result in local pH changes, which could be unfavorable for biological particles. To suppress the electrode reactions, an alternating current (AC) electric field can be used for particle focusing, often at a frequency of around 1 kHz. Under these conditions, linear electroosmosis and electrophoresis are suppressed due to the alternating direction of the electric field over time. Instead, dielectrophoresis (DEP) and alternating current electroosmotic flow (AC-EOF) are the dominant mechanisms described in literature^16,17^ that can result in the focusing of particles.

Dielectrophoresis can be understood by considering that in an AC electric field, materials align their dipole moment with the electric field. For a particle in a fluid medium, the ratio between the polarizability of the fluid and the particle, known as the Clausius-Mossotti factor determines whether the fluid or particle dipole behavior is dominant. For a particle in a uniform electric field, the electric field acts with the same force magnitude on both poles and consequently, the particle is not affected by the electric field. However, in a nonuniform electric field, for instance between 2 electrodes of unequal size, the dipole force on the poles of the particle is different and consequently, dielectrophoresis moves the particle either toward the electric field minimum or maximum depending on the Clausius-Mosotti factor.^18^ AC-EOF occurs when the electrodes are positioned in such a way that there is a nonzero component of the electric field parallel to the electrode surface, which is for instance the case for coplanar electrodes. In this case, the electric field affects the movement of the counterions inside the double layer of the electrode, which results in a stationary fluid flow. The magnitude of the AC-EOF flow is next to electric field component along the electrode also determined by other factors such as for instance the frequency of the electric field and the conductivity of the salt solution.^16,19^ AC-EOF is explained in more detail in the section Theory.

**Figure 1:**
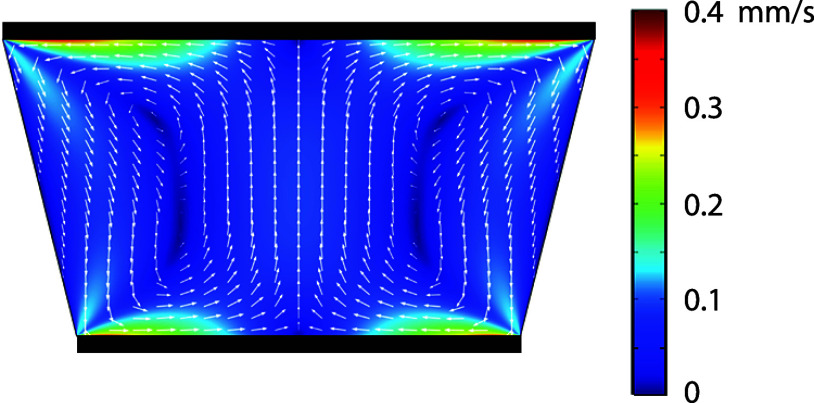
Numerical simulation of a chip geometry with an acute angle of 76° and the AC-EOF induced vortex flow profile of the fluid for a frequency of 1 kHz and a voltage amplitude of 1 V. The black bars indicate the positions of the electrodes.

Recently, it was shown by Tiflidis et al.^20^ and Westerbeek et al.^21^ that applying an alternating current across a rectangular microfluidic channel with deformed insulating side walls, results in distinct vortices due to AC-EOF. It was shown that the shape of the walls determines the structure of the vortices generated. While that study was mainly focused on reducing the axial dispersion in microfluidic chromatography, it showed that presumably in the absence of pressure-driven flow through the channel, the particles moved from the channel walls toward the centerline of the channel. It was postulated in several studies that this is caused by AC-EOF vortices.^20−22^ An example of such a vortex flow pattern is illustrated in Figure 1 for a trapezoidal channel geometry.

**Figure 2:**
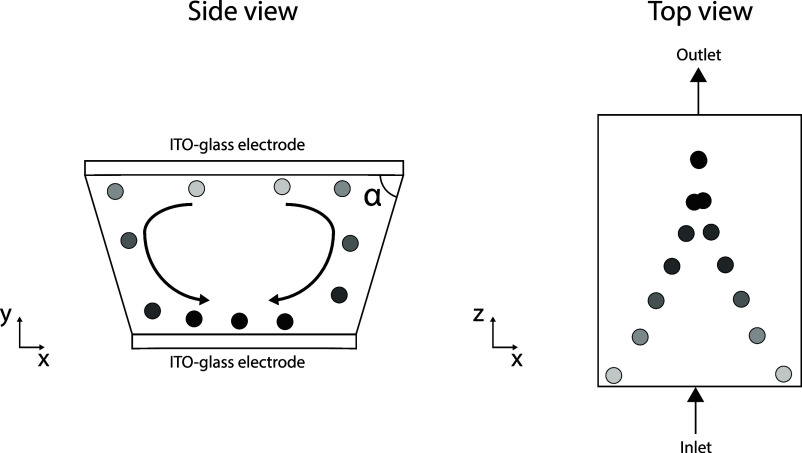
Schematic side and top view of the chip with 2 possible particle paths where the light particles show the initially unfocused particles, while the darker colored particles show the same particle at later times, indicating the movement toward the focused position. Angle α is the angle that is referred to as the acute angle in this work.

In a conference contribution of Tiflidis et al.,^22^ it was also observed that when flowing a dilute particle suspension through a microchannel, the particles are focusing along the centerline of the channel under the influence of an AC field. In the case of a trapezoidal geometry oriented with the widest side a the top, it is expected that based on the resulting AC-EOF flow pattern the particles move from any position toward the bottom and the centerline of the channel, as schematically illustrated in Figure 2.

This phenomenon could potentially be exploited as an interesting particle focusing method to complement other available techniques. Since it is a relatively simple chip design that only requires a slightly deformed wall and two electrodes, it may have an advantage over more intricate particle focusing devices.

However, the proposed AC-EOF vortices can only partially explain the observed particle focusing, since counter-rotating vortices cannot cause the trapping of particles along the center of the channel but instead would result in continuous recirculation of particles. This implies that an additional force would need to act on the particles to keep them focused. In literature, similar experiments between parallel electrodes with an AC electric field have been performed with somewhat larger than 1-μm particles in the absence of a pressure-driven fluid flow.^23−25^ For these systems, it was argued that the particles can attain a stable equilibrium position by a combination of gravitational force toward the electrode and an electrohydrodynamic force that repels particles from the electrode. In some of these experiments also long-range asymmetric electric fields (AREF) effects^25^ could play an important role in the equilibrium position. While these provide plausible explanations for those experiments, it is very unlikely that any of these effects would be able to overcome the upward hydrodynamic force of the AC-EOF vortices in our case due to the small density difference between the polystyrene particle and water and the small particle size means that the effect of gravity can be neglected as the sedimentation velocity is in the order of 10 nm/s while the resulting AC-EOF velocities are close to 0.1 mm/s. The nearly equal cation and anion diffusion coefficients of the salt used in our experiments (KCl) limits the magnitude of both diffusiophoresis and AREF effects. To understand how the particle focusing is nevertheless observed in this experimental system, a series of experimental and theoretical studies were carried out to clarify the observed particle focusing behavior.

## Theory

### AC-Electroosmosis

AC-electroosmosis (AC-EOF) arises when an electric field bends between electrodes, which results in a component of the electric field parallel to the electrode surface. In a system with deformed or nonperpendicular walls, this occurs close to the corners of these walls. Although the electric field alternates in polarity, at sufficiently low frequencies (depending on the concentration below several MHz up to GHz^26^) the double layer can still form and is in quasi-equilibrium. Since the double layer contains primarily ions charged opposite to the polarity of the electrode, the motion of these ions due to the electric field results in an effective fluid flow. The polarity of an electrode in an AC-field switches in time. This affects both the type of ion that is primarily present in the double layer (cations or anions) and the direction of the electric field. Since both these factors change over time, this results in a stationary flow despite the oscillating electric field. The AC-EOF slip velocity on the electrode can be described by the equation:^16^
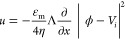
1where Λ is the ratio between the potential drop over the diffuse part of the electrostatic double layer and the potential drop over the complete electrostatic double layer, which was assumed to be 0.5, equal to the value assumed by Tiflidis et al.,^20^ but values as low as 0.2 have been found.^16^ ϕ is the potential located just outside the electrostatic double layer and *V*_*i*_ is the potential applied to the corresponding electrode. ε_m_ is the permittivity of the medium and η is the viscosity of the medium.

### Induced Dipole Force

In response to an AC-electric field the charges in a dielectric particle become polarized and result in a dipole. Depending on the frequency of the electric field, this is for instance caused by the movement of the ions in the double layer of the particle in response to the applied electric field up to the electrolyte relaxation frequency of several MHz. Above this frequency, the particle’s charge is polarized by the difference in polarizability of the molecules in the medium and particle surface.^26^ When such a polarized particle is located in the vicinity of a conducting plane, it induces opposite charges on the electrode that result in an attractive force toward the electrode irrespective of the polarizability of the particle and the medium. The magnitude of this force can be calculated based on Coulomb’s law, by considering that the charge induced on the electrode is located inside the electrode at an equal distance from the electrode surface relative to the location of the particle above the electrode surface. This calculation method is known as the method of images and for a dielectric sphere near a conducting plane the force can be calculated by^27^
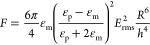
2where ε_m_ and ε_p_ are the permittivity of the medium and the particle, respectively. *E*_rms_ is the root-mean-square electric field strength undisturbed by the particle, *R* is the radius of the particle and *h* is the distance from the wall to the center of the particle.

While this expression is only valid for particles when *h* ≫ *R*, it illustrates that regardless of the magnitude of the dielectric properties of the particles and the medium, the force associated with the induced dipole between the particle and the electrode is always attractive. Due to the limited range of applicability of eq 2, the induced dipole force will be evaluated numerically based on simulations similar to the implementation of Pérez and Fernández-Mateo.^27^ From these simulations, the induced dipole force is calculated using the Maxwell stress tensor without any further use of eq 2. More details on the simulations can be found in the Material & Methods section. Finally, it should be pointed out that although the underlying physics are similar to those observed in dielectrophoresis, in literature the term dielectrophoresis is mostly used for the movement of particles in a distorted electric field. However, in the channel, the electric field is only distorted near the side walls of the channel, while the focused particles are located near the centerline of the channel where the electric field is relatively uniform. Therefore, the force on the particle is not governed by the nonuniformity of the applied electric field. For this reason and because the particle is always attracted to the electrode regardless of the Clausius-Mossoti factor, we chose to refer to this interaction as the induced dipole interaction instead.

## Material & Methods

### Particle Focusing Experiments

The microfluidic devices were made in-house following the procedure described in Tiflidis et al.^20^ In brief, the devices were made from fused silicon wafers that were sputtered with a 70 nm indium tin oxide layer (ITO), which acts as a transparent electrode. Two of these wafers were bonded together with a 20 μm thick epoxy foil (NC-S0075A-F, Tokyo Ohka Kogyo Co.) The epoxy foil defines the side walls of the chip that bent slightly during the bonding process. The access holes for fluid entrance and exit were created by powder blasting. Both wafers were put on top of each other with an offset to make sure that the ITO electrode inside the chip could be connected to electrical connections using an in-house-built chip holder.

For the particle focusing experiments, 0.0025 wt % of 1.14 μm fluorescent polystyrene particles were used (Fluored,Microparticles GmbH.) in 0.1 mM KCl solution (Sigma-Aldrich, > 99.5%). For the fluid connections glass capillary tubing (Postnova Analytics, ID:20 μm) was used. The fluid was pushed through the chip by a pressure system that applies maximally 1 bar (Fluigent). A flow controller (Fluigent, size M) was connected to the outlet of the chip. Before each measurement, a maximum pressure difference of around 1 bar was briefly applied to make sure all the air was removed from the system, without controlling the flow rate. Next, the flow controller was set to a flow rate between 0.1 and 2 mL/min, which corresponds to average fluid velocities between 2.1 and 6.3 mm/s. When the fluid flow had stabilized, an AC sine wave was applied to the electrodes with an amplitude of 1 V (peak-to-peak potential of 2 V) at a frequency of 1 kHz with a frequency generator (Techtronix AFG, AFG2021). To make sure that most of the frames contained the onset of the particle focusing behavior, the recording of the video was started simultaneously with the electric field. The videos were recorded at a frame rate of 600 fps for 10 s thereafter the electric field was switched off.

The particle movement was tracked by Trackmate, a tool within the freely available ImageJ image analysis software.^28^ Next, the velocity was extracted from the videos using an in-house written Matlab script based on the distance traveled between two frames. To calculate the average velocity profiles across the width of the channel at each flow rate, the width and height were split up into bins of 2 and 10 pixels respectively and the average was taken along the length of the channel, to obtain a single velocity along the width. This procedure was performed for a total of three experiments per flow rate and then the ensemble average of the velocity was calculated as well as the standard deviation along the width of the channel.

### Simulations

The AC-EOF simulations were performed in 3D by Comsol solving the dimensionless Laplace equation, which calculates the potential drop over the bulk electrolyte. From the potential distribution, the slip velocity at the electrodes is calculated and the resulting fluid flow is obtained by the Navier–Stokes equations as described originally by Green et al.^16^ and was implemented in the same way as described in Tiflidis et al.^20^ Our simulations differ in that the geometry is extended from a 2D shape into a 3D shape, where the 2D shape is extruded by 10 μm to ensure that the profile in the center part of the channel is not influenced by the no-slip conditions of the additional walls of the 3D geometry. It was found that the hydrodynamic drag based on Stokes’ law, which assumes the particle is a point underestimates the hydrodynamic force for a particle close to the bottom electrode (see Supporting Info S4). Therefore, a sphere with a diameter of 1 μm was inserted in the channel geometry to represent a focused particle. For this simulation the physics were not solved inside the spherical domain, making the “particle” effective completely electrically insulating and applies a no-slip condition at the edge of the sphere. The particle was positioned in the center of the channel geometry along the width (*x*-direction) and the depth (*z*-direction) of the channel geometry to represent a focused particle. For each simulation the particle was fixated at a specific gap distance varying between 100 nm and 2 μm between the bottom electrode wall and the particle edge. From these simulations, the hydrodynamic force acting on the particle in the *y*-direction was calculated by numerically integrating the hydrodynamic stress tensor over the surface of the particle, which is a built-in calculation in Comsol. In all these simulations, the particle was assumed to be impenetrable by the electric field and a no-slip condition was assumed on the surface of the particle. In addition, simulations without a particle were performed. All simulations were solved at an AC frequency of 1 kHz, an electric potential amplitude of 1 V, and a solution conductivity that matches a salt concentration of 0.1 mM KCl, which is similar to the experimental conditions.

Since eq 2 can only be applied to calculate the induced dipole force in case of a dielectric particle in a dielectric fluid positioned far away from a conducting surface, the induced dipole force on a particle is evaluated based on similar axial symmetric numerical simulations as described by Pérez and Fernández-Mateo^27^ for various particle-wall gaps. The main difference between our simulations and the simulations described in literature is that we do not consider a third electrode material domain. This simulation consists of a relatively large axial symmetric cylindrical domain with the spherical particle inserted at various gap distances from the bottom electrode between 100 nm and 2 μm. In these simulations the Laplace equation is solved for the entire domain via the Electrostatics module in Comsol. These simulations were performed for the case of a dielectric particle (ε_p_ = 2) and a particle with a high dielectric constant (ε_p_ = 8000), mimicking the behavior of a conducting particle, because the dielectric constant is much higher compared to the surrounding fluid (water ε_m_ = 80), resulting in a nearly constant potential in the particle. These cases are solved for illustrative purposes since these are the two limiting cases for induced dipole attraction. In these simulations, the electric field strength was obtained from the AC-EOF simulations and was used as a boundary condition at the top of the cylindrical domain, while a ground boundary condition was used at the bottom of the cylindrical domain. At the outer radial edge a zero charge condition was applied, while a symmetry condition was used along the centerline of the axial symmetric cylindrical domain.

A third case considers the effect of surface conductance on the surface of the particle on the induced dipole force. This simulation was performed by using the Electric Currents module in Comsol, which includes the effect of the conductivities next to the permittivity of the particle and the medium on the electric field. In this simulation, the surface conductance effect was included by the Electric Shielding boundary condition, where the particle was assumed to have an electrical conductivity of 0.004 S/m, which corresponds for a 1 μm particle, to a surface conductance of 2 nS, which is calculated in Comsol by multiplying the conductivity with a particle “thickness” equal to the particle radius. This is in line with literature values.^29^ The fluid was assumed to have a conductivity of 1.5 mS/m, corresponding to a salt concentration of 0.1 mM KCl. The conductivity of the bulk of the polystyrene particle was assumed to be very low and a value of 10^–5^ nS/m was used. In this case, the applied potential at the top boundary condition was chosen to closely match the electric field strength of the AC-EOF simulations. In all the above-described induced dipole simulations the induced dipole force is evaluated by calculating the Maxwell stress tensor in the *y*-direction, which is built-in calculation in Comsol.

## Results & Discussion

**Figure 3:**
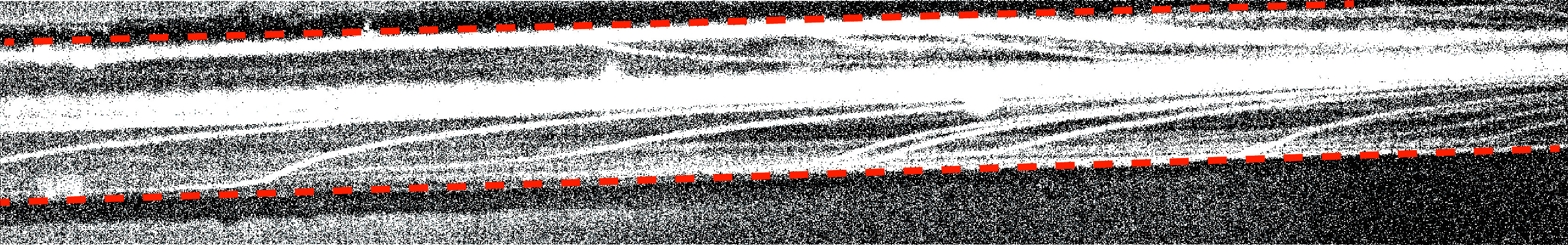
Composite image overlaying multiple video frames from the video where the microscope was focused at the bottom of the chip under an applied AC-field of 1 kHz with an amplitude of 1 V. The fluid flows from left to right and the dashed line indicates the approximate position of the side walls.

**Figure 4:**
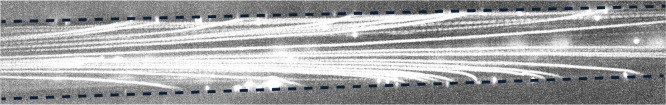
Composite image image overlaying multiple video frames from the video where the microscope was focused at the top of the chip under an applied AC-field of 1 kHz with an amplitude of 1 V. The fluid flows from left to right and the dashed line indicates the approximate position of the side walls.

Figures 3 and 4 show composite images of all the particle positions within the first 100 frames of an experiment. This image represents the trajectories that the particles follow. In Figure 3 the microscope was focused on the bottom of the microchannel. This figure shows a bright stripe at the centerline of the chip, which is where most fluorescent particles will eventually be focused. The more faint streaks show the particle trajectories of the unfocused particles. All these trajectories have in common that they start near the side walls and converge toward the centerline of the channel further downstream. In Figure 4 the microscope was focused on the top of the chip. In this figure, most of the particle trajectories diverge toward the closest sidewall. Furthermore, it can be seen that when the particle is initially positioned closer to the wall, the pathway to the side walls is shorter compared to the particles closer to the centerline. This indicates that the fluid velocity along the *x*-direction of the chip is larger near the edges of the chip compared to the centerline. These results indicate that the AC-EOF flow profile is very likely to consist of 2 counter-rotating vortices that move the particles from the top via the side walls toward the centerline of the channel at the bottom. The above-described particle movement can also be observed in the videos which can be found in the Supporting Material.

As discussed in Tiflidis et al.^20^ and shown in Figure 1, a trapezoidal geometry results in two counter-rotating vortices by AC-EOF. Furthermore, the cross-sectional SEM images of the chip were inconclusive on the precise geometry (see Supporting Info S1). Therefore, we assumed in the numerical model that the microchannel has a trapezoidal cross-section. To study the influence of the acute angle, we modeled three different geometries with acute angles (α) of 76°, 82°, and 88°. The numerical 2D velocity profile of AC-EOF flow in a trapezoidal channel cross-section is shown in Figure 1. In this figure, two distinct vortices can be observed, which flow toward the sidewalls at the top and toward the centerline at the bottom. Furthermore, it is observed that at the top and bottom, the magnitude of the velocity increases toward the sidewalls. This is in qualitative agreement with the particle movement observed in Figures 3 and 4.

**Figure 5:**
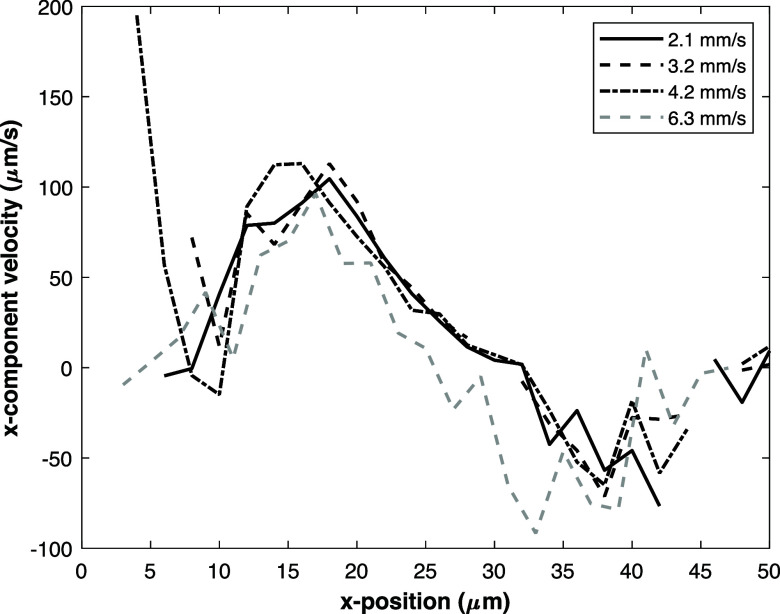
Experimental *x*-component velocity (focusing velocity) along the *x* position in the channel for various superficial fluid velocities obtained from videos with the microscope focused on the bottom of the microchannel. At the positions where some lines are missing not sufficient data was obtained.

To show that the focusing velocity is not affected by inertial focusing effects of the fluid flow velocity (along the *z*-direction), as was suggested by Tiflidis et al.,^22^ the particle focusing velocity was inferred at different fluid flow velocities (see Figure 5). This figure shows that there is no apparent effect of the pressure-driven fluid flow on the focusing velocity of the particles. Another strong indication that the focusing is primarily governed by AC-EOF is that the velocity is the highest near the side walls of the microchannel and decreases toward the center, which was also observed previously in simulations (Figure 1) and in experiments (Figure 4).

**Figure 6:**
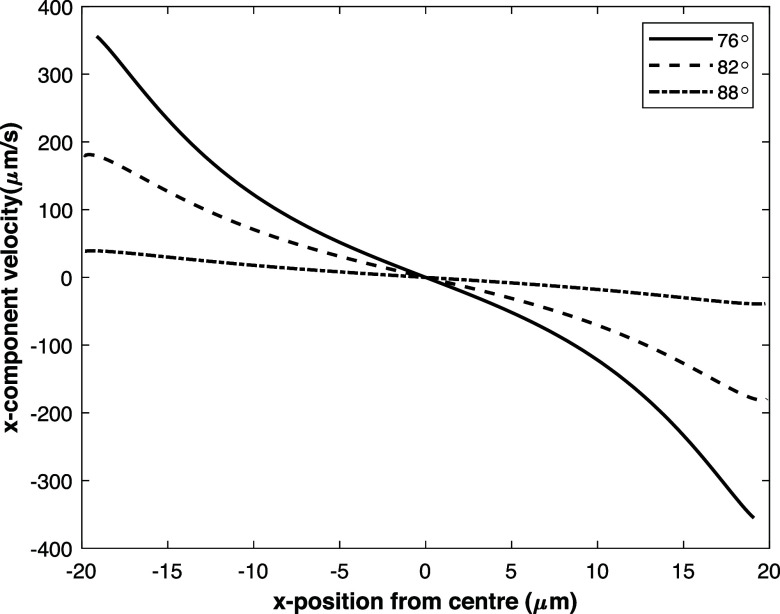
Focusing velocities based on numerical 3D simulations evaluated at the bottom electrode wall for geometries with an acute angle of 76°, 82°, and 88°.

The qualitative trend of the numerical focusing velocity is similar to the experimentally determined profile where the velocity is the highest toward the sides of the channel and drops toward zero at the centerline (see Figure 6). This figure shows that as the acute angle approaches a straight wall (at 90°), the focusing velocity drops significantly as this reduces the electric field bending over the electrode surface. The maximum focusing velocity of a geometry with an 88° angle is theoretically about 30 μm/s, which highlights that even a slightly slanted wall, can still lead to a significant focusing velocity, by considering that the microchannel is only 20 μm wide. Finally, by comparing the experimental and numerical results it can be inferred that the microchannel used during our experiments can be best described by a perfect trapezoidal geometry with an acute angle between 82° and 88°. However, as noted previously, the actual geometry of the microchannel is probably more complex than a trapezoidal geometry. For this reason we did not try to find a geometry where the numerical and experimental results match more closely.

**Figure 7:**
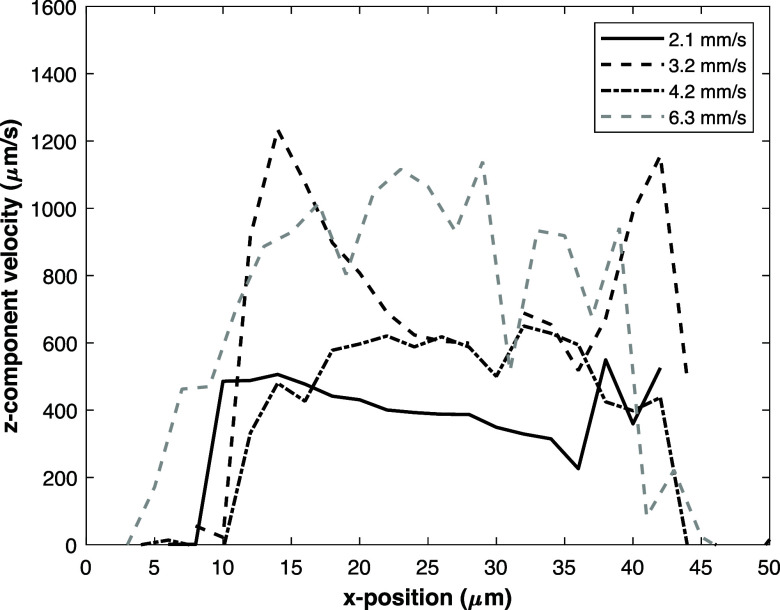
Experimental z-component velocity profile of the particles along the x-position in the channel for various average fluid flow velocities tracked near the bottom electrode. At the positions where some lines are missing not sufficient data was obtained.

The experimentally determined axial velocity component of the particles near the bottom electrode (in the *z*-direction) is scaling approximately with the average fluid velocity (see Figure 7) calculated from the set flow rate and the cross-sectional area, assuming the effect of the deformed side walls is negligible. In this figure, it can be seen that the measured velocity of the particles is much lower than the average fluid velocity. This indicates that the particles are in close proximity of the bottom electrode wall. The reason for this is that only the particles that are focused or the particles that are eventually driven toward the centerline near the bottom electrode move slowly enough to track them and quantify their velocity at a frame rate of 600 fps. For this reason and because these particles move relatively quickly toward the centerline of the channel, the sampling of the *z*-component velocity data from positions other than at the centerline of the channel is limited. Despite some peculiar behavior of the averaged velocity profile at a fluid velocity of 4.2 mm/s, overall, the particles seem to move the fastest at the centerline and more slowly toward the sides of the channel which is in line with an ordinary flow profile in a duct. Considering that the Reynolds number of the fluid is small and the particles are also relatively small, it is expected that the particles have negligible inertia. The particle distance from the bottom electrode wall can in principle be estimated by calculating the position where the particle’s velocity matches the fluid velocity profile. However, our more detailed analysis provided in Supporting Information Section S2 showed that the increased friction between the particle and fluid should be taken into account because the focused particles are very close to the wall. Therefore, the particle position was estimated by using the lubrication theory based on the approximation of Pasol et al.^30^ It was found that based on the velocity of the focused particles along the *z*-direction of the channel, the gap between the focused particles and the bottom electrode wall was roughly between 10 and 100 nm.

As mentioned previously, the vortices caused by AC-EOF can only partially explain the particle focusing phenomenon. Since in the absence of other forces acting on the particle, the particle would be lifted from the bottom back toward the top of the channel (see Figure 1). A potential attractive force between the electrode wall and the particle concerns the induced dipole force. Therefore, a quantitative force balance between the hydrodynamic and the induced dipole forces was constructed, based on numerical simulations.

**Figure 8:**
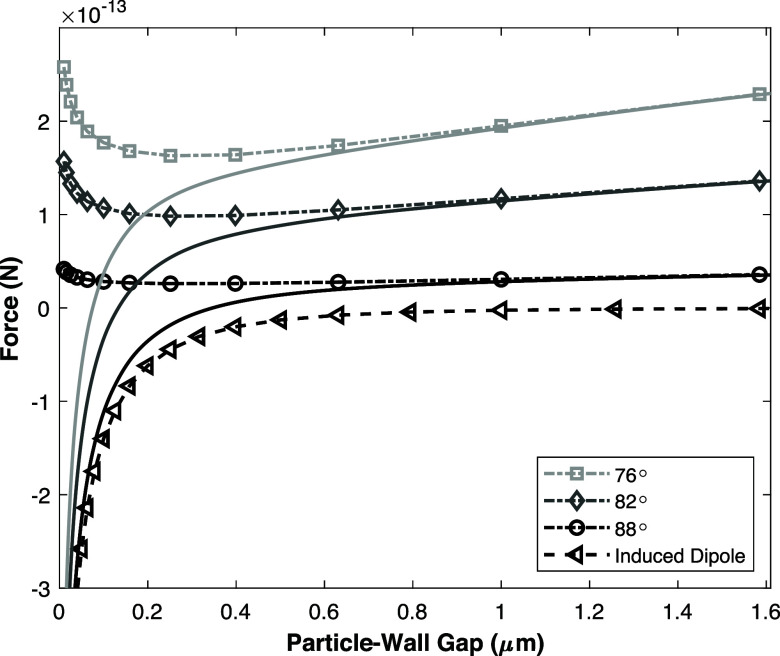
Force balance based on numerical simulations for the repulsive hydrodynamic force for different acute angles and the attractive induced dipole force assuming a particle with a diameter of 1 μm with surface conduction with lines to guide the eye. The solid net force curves are based on spline interpolations of the forces.

In Figure 8, the theoretical hydrodynamic repulsion force and the induced dipole force as well as the resulting net force are presented as a function of the particle-wall distance. In this figure, it can be seen that for all geometries, when the gap between the particle and the wall is more than 0.3 μm, the hydrodynamic force is stronger and consequently the particle will be pushed away from the wall. For a gap smaller than 0.3 μm, the behavior depends on the exact geometry of the channel, since for sharper angles the AC-EOF flow is stronger, and consequently, the hydrodynamic repulsive force is stronger in the center of the chip. For a gap of about 100 nm, the induced dipole force is dominating for all geometries presented. This results in a net attractive force between the wall and the particles and this would lead to the particles sticking to the bottom electrode. It should be noted that the induced dipole force also depends on the contrast of conductivity and permittivity between the particles and the medium. Therefore, the limiting cases for a purely dielectric and conducting particle are presented in the Supporting Information Section S3 to provide more insight in the range of magnitudes of the induced dipole force.

Based on the experimentally observed focusing velocity it was estimated that the chip walls have an acute angle between 82° and 88°. Therefore, it is likely that at the sides of the chip, the particles are transported by the vortices to a distance within approximately 0.3–0.2 μm from the bottom wall, where they cannot escape by the hydrodynamic repulsive force once they arrive at the bottom and centerline of the channel. The attractive induced dipole force can explain why the particles do not flow upward due to the AC-EOF flow profile at the centerline of the channel. However, the induced dipole force alone cannot explain the experimental focusing height of the particle, which was estimated to be between 10 and 100 nm, because the hydrodynamic and induced dipole forces do not result in a stable equilibrium position. At any position the particle is either repelled (hydrodynamic) or attracted (induced dipole) to the electrode. Therefore, we will now propose some additional repulsive forces that could potentially explain our experimental observations.

The electrostatic double layer interactions between the charged wall and particle can have a pronounced effect. Due to the low salt concentration of 0.1 mM, the Debye length is about 30 nm. This means that for a distance of 60 nm up to 0.2 μm the electrostatic interaction could still be significant. Fagan et al.^31^ found that for particles in an AC electric field (5 V peak-to-peak, KOH solution) at frequencies close to 1 kHz, the particle height reduces by about 20% compared to the case without any field. Therefore, it is likely that the electrostatic force is not influenced considerably by the applied AC-field. As a first approximation, the equilibrium DLVO forces (electrostatic repulsion and Lifshitz- van der Waals attraction) were evaluated in addition to the hydrodynamic and induced dipole forces.^31^

**Figure 9:**
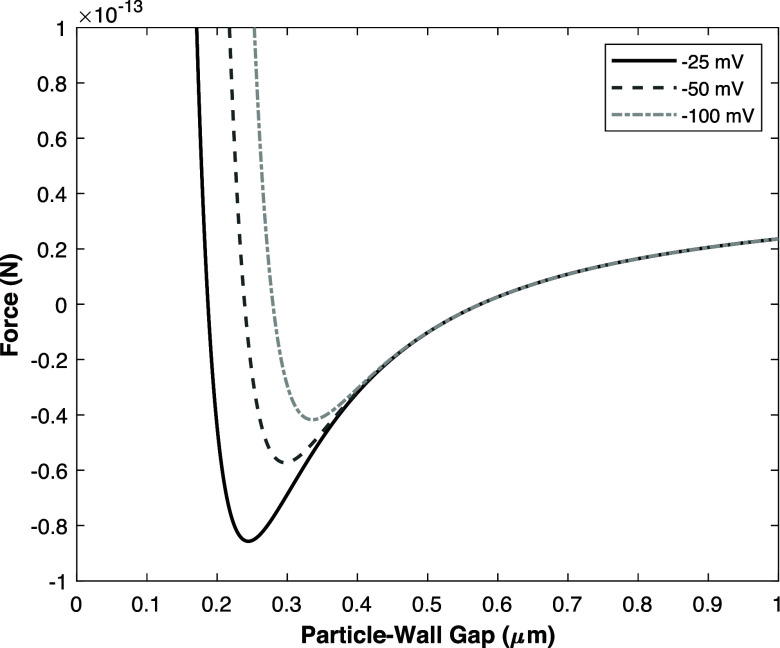
Total net force on a metallic particle for an 88° chip geometry based on numerical simulations including induced dipole, hydrodynamic repulsion and equilibrium electrostatic repulsion for 3 different zeta potentials for the particle and wall.

It should be noted that the equilibrium electrostatic force is relatively strong when the particle is within a distance of 0.2 μm compared to the induced dipole and hydrodynamic force. Only in the case of a metallic or highly conductive particle in a channel with an acute angle of 88° the induced dipole is strong enough to result in a stable equilibrium position (see Figure 9), while for the other cases, the particle would be repelled from the surface at any distance from the electrode, by a combination of the electrostatic and hydrodynamic forces. While this restriction makes it difficult to link the theoretical results directly to the experimental results since experimentally polystyrene particles were used that are nonconductive in the bulk material. Nevertheless, it can still provide an estimate as to what extent electrostatic interactions are significant.

In Figure 9, the aforementioned total force is depicted for metallic-like particles, assuming various different zeta potentials for the electrode wall and particle. At a small zeta potential of only 25 mV, the equilibrium position of the particle is located at a gap of approximately 0.25 μm, while at an extreme zeta potential of −100 mV the equilibrium position is approximately at 0.3 μm. From the experiments, the particle-electrode gap was estimated to be maximally around 0.1 μm. Therefore, it is unlikely that even by assuming the induced dipole force of a conductive particle the equilibrium electrostatic force is, without corrections for the alternating field, sufficient to complete the particle focusing force description. For instance, as the applied potential and thus the zeta potential of the electrode varies in time, the effective electrostatic repulsion acting on the particle may be reduced. Despite this, the electrostatic repulsion could still be a relevant force that should be taken into account in future work.

Finally, it should be noted that the electrostatic interaction between an electrode and a particle in an AC-field can be rather complex and could for instance also be influenced by strong coupling of the ions to the electrode surface.^32^ This would result in a reduction of the electrostatic repulsion. It could also lead to osmotic pressure repulsion even when the electrode and particle are oppositely charged.^33,34^

The electrode reactions, which have been neglected so far, could also play a significant role. While it is often assumed that Faradaic reactions do not occur at frequencies close to 1 kHz, Wang et al. showed that even at 1 kHz, the pH can change to some extent up to several tens of micrometers away from the electrode for a peak to peak potential of 5 and 2 V at a lower frequency of 100 Hz.^35^ While this is not necessarily the same as our experimental conditions, it indicates that it may be too simplistic to completely rule out the effect of electrode reactions, since the resulting pH gradient could result in a repulsive diffusiophoretic displacement of the particles.^35^ Another effect that could result in a repulsive force as a result of electrode reactions was proposed recently by Jarvey et al.^36^ In their numerical study, it was found that including Faradaic reactions can result in an asymmetric rectified electric field effect, even in case the diffusion coefficients of both ions are nearly identical. This gives rise to an electric field due to the mismatch in the transport of ions, which results in the transport of particles away from the electrode.

All these forces could potentially prevent the particle from sticking to the electrode as a result of the induced dipole force. However, based on the set of experiments and simulations performed so far, it is not yet possible to determine what the most dominant contribution is that prevents the particles from sticking to the electrode wall.

## Conclusions

In this work, we present a potential new continuous particle focusing method by applying an alternating current electric field in a rectangular microchannel with slightly slanted side walls. Furthermore, we provided the start of a theoretical basis to rationalize the observed particle focusing behavior based on AC-EOF and induced dipole interactions. Due to the slanted side walls, an AC-EOF flow results in a trapezoidal channel geometry to a pair of counter-rotating vortices. These vortices transport the particles toward the bottom and centerline of the channel, where the particles are focused within a distance of about 0.2 μm from the electrode. This is the distance where the (numerical) hydrodynamic force is smaller compared to the induced dipole attraction. While it is at this stage still unknown what specific repulsive force prevents the particles from sticking to the wall, we hypothesized 3 potential candidates: electrostatic repulsion, diffusiophoresis due to electrode reactions and asymmetric rectified electric field forces due to the electrode reactions. The induced dipole attraction depends on the dielectric and conductive properties of the particles. Therefore, our particle focusing technique can potentially be a promising technique for sorting mixtures of particles with different dielectric properties. However, further experiments with particles and particle mixtures of different size and material need to be performed in a dedicated microfluidic device that allows to separate the focused particles from the unfocused particles to determine the efficiency of this particle focusing method. Furthermore, it would be interesting to perform experiments in channels with better-defined side wall geometries, which could for instance be achieved by 3D printing techniques, as demonstrated for instance by Partiban et al.^37^ and Convery et al.^38^

## Supporting Information Available

S1: cross-sectional SEM images of the microchannel; S2: detailed analysis of the experimental particle height; S3: evaluation of the induced dipole force of a metallic and purely dielectric particle; and S4: comparison between the Stokes force of the fluid and the actual hydrodynamic force acting on a particle from simulations (PDF)
Video S1: video of particle focusing with microscope focused at bottom of the channel for a fluid flow of 0.2 μm/min0 (AVI)
Video S2: video of particle focusing with microscope focused at bottom of the channel for a fluid flow of 0.1 μm/min (AVI)
Video S3: video of particle focusing with microscope focused at the top of the channel for a fluid flow of 0.2 μm/min (AVI)

## Supplementary Material

la4c02135_si_001.pdf

la4c02135_si_002.avi

la4c02135_si_003.avi

la4c02135_si_004.avi
