# Supplementary material for: Continuous Focusing of Particles by AC-Electroosmosis and Induced Dipole Interactions
Source: Langmuir. 2024 Sep 13;40(38):19988–96. doi: 10.1021/acs.langmuir.4c02135 (PMC11428184; doi:10.1021/acs.langmuir.4c02135)
Supplement: Supplementary file 1 — la4c02135_si_001.pdf [file la4c02135_si_001.pdf]

Supporting Information

Continuous Focusing of Particles by AC-Electroosmosis and Induced Dipole Interactions

Harm T.M. Wiegerinck,[*,†] Jeffery A. Wood,[†] Jan C.T. Eijkel,[‡] Rob G.H. Lammertink,[†] Itzchak Frankel,[¶] and Antonio Ramos[§]

†*Soft Matter, Fluidics and Interfaces, MESA+ Institute for Nanotechnology, University of Twente, 7500AE Enschede, The Netherlands*

‡*BIOS/The Lab-on-a-Chip group, MESA+ Institute for Nanotechnology, University of Twente, P.O. Box 217, AE Enschede, The Netherlands*

¶*Department of Aerospace Engineering, Technion - Israel Institute of Technology, Haifa 32000, Israel*

§*Departamento de Electronica y Electromagnetismo, Universidad de Sevilla, Avenida Reina Mercedes, s/n 41012 Sevilla, Spain*

E-mail: harmwiegerinck@gmail.com

number of pages: 11

number of figures: 5

Contents

S1 SEM Images **S3**

S2 Analysis of the Particle-Electrode Gap in the Middle of the Chip **S4**

S3 Force Balance with Different Particle Polarizability **S8**

S4 Hydrodynamic Force versus Stokes Force **S9**

References **S11**

S1 SEM Images

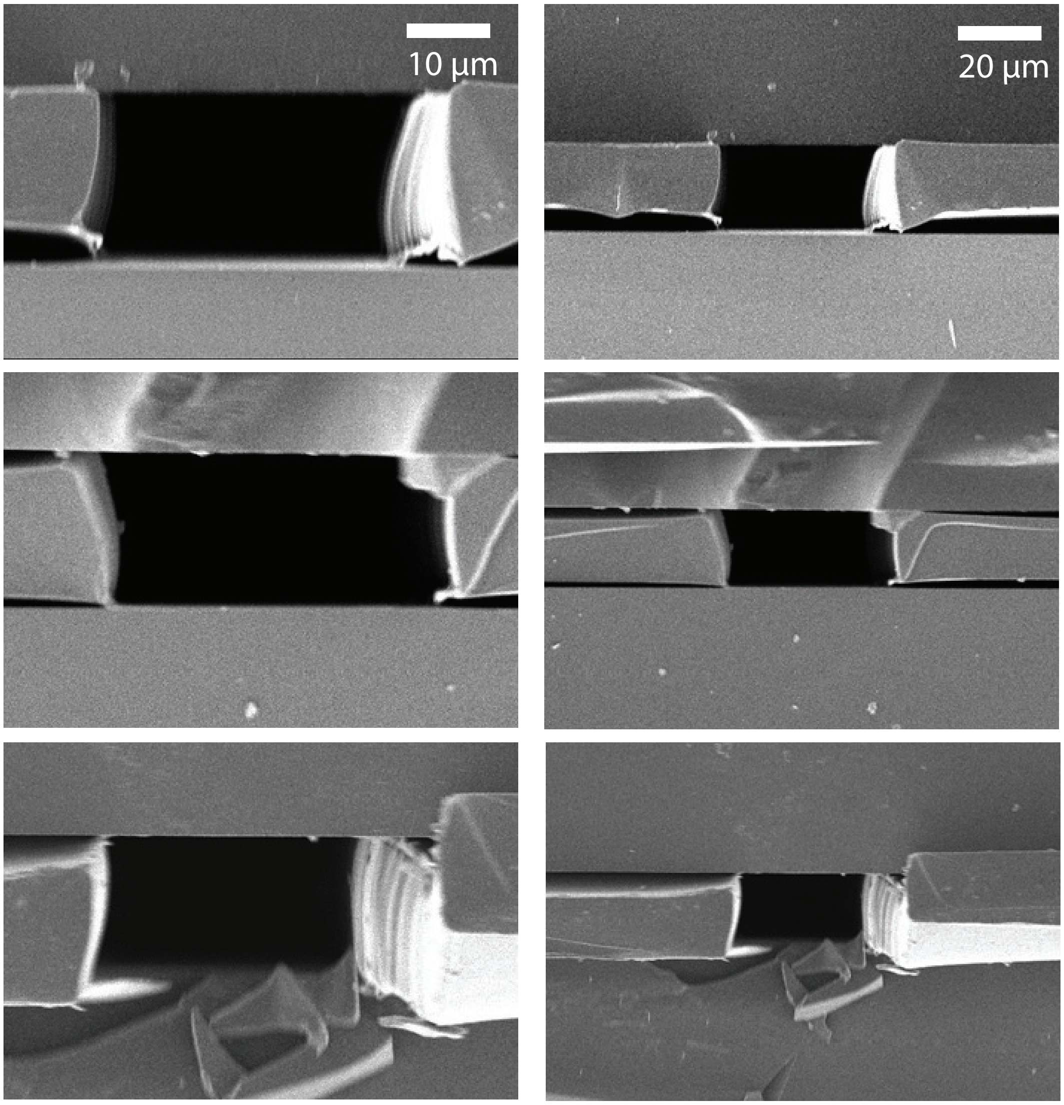

Figure S1: 3 different cross-sections of particle focusing chips magnified 2000x and 1000x.

The SEM images of the cross-sections of the chips clearly show that the foil walls are slanted (see Figure S1). However, since all the 3 cross-sections look somewhat different and because of the delamination of the foil with the wall, it is not possible to define a consistent chip geometry from these results. Therefore, it was chosen to assume a simple trapezoidal geometry for the numerical simulations and the trapezoidal acute angle was varied between 76 and 88 degrees to study the effect of shape on the resulting focusing velocity .

S2 Analysis of the Particle-Electrode Gap in the Middle of the Chip

To analyze the distance of the focused particles from the electrode it is initially assumed that the particles follow the fluid perfectly, because of the low Reynolds numbers and the small particles. The velocity profile of a rectangular duct with straight side walls can be numerically determined by a Fourier series derived by Bousinesq:[1,2]

$$u(x,y) = \frac{G}{2\mu} y(h-y) - \frac{4Gh^2}{\mu\pi^3} \sum_{n=1}^{\infty} \frac{sinh(\beta x) + sinh\left(\beta\left(l-x\right)\right)}{sinh(\beta x)} sin(\beta y) \quad \text{(S1)}$$

where β is given by:

$$\beta = \frac{(2n-1)\pi}{h} \quad \text{(S2)}$$

where G is the pressure drop, μ is the dynamic viscosity, y is the position along the height in the channel, h is the height of the channel, l is the width of the channel and x is the position along the width of the channel. The pressure drop G is initially unknown. However it can can be determined by an analogous equation which relates the flowrate to the pressure drop, which allows calculating G by:

$$G = Q/C \quad \text{(S3)}$$

Where Q is the volumetric flowrate and the geometric constant C is defined by:

$$C = \frac{h^3 l}{12\mu} - \frac{16h^4}{\pi^5\mu} \sum_{n=1}^{\infty} \frac{1}{(2n-1)^5} \frac{cosh(\beta l) - 1}{cosh(\beta l)} \quad \text{(S4)}$$

From this, the velocity profile can be calculated for a rectangular channel under the assumption that the effect of the slanted side walls on the fluid velocity profile is minor.

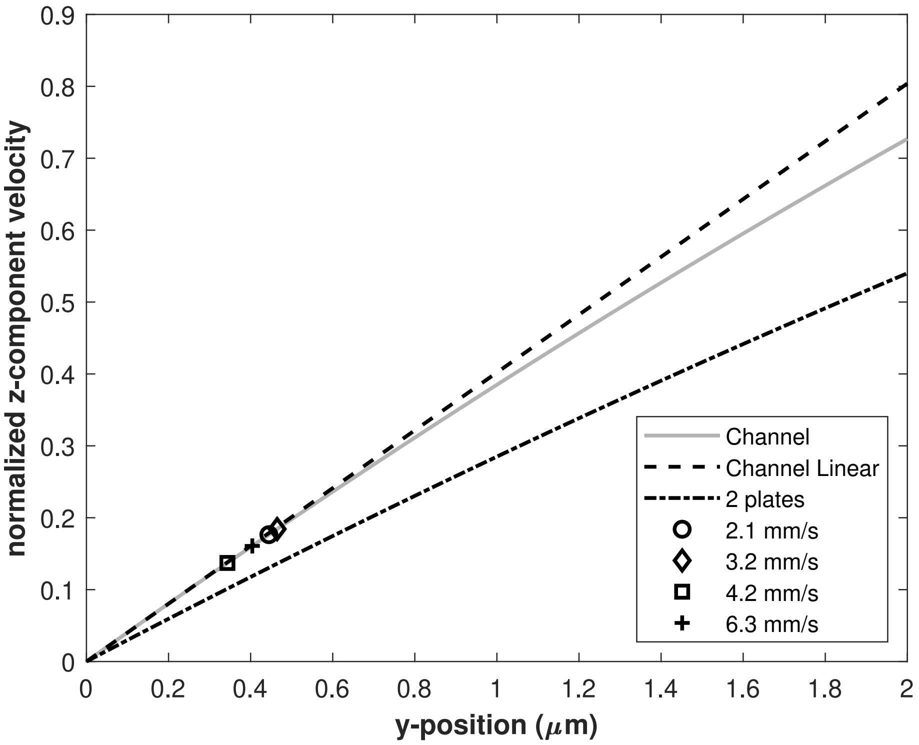

Figure S2: The fluid normalized velocity in the middle of the channel near the electrode wall, with the markers indicating the approximate normalized velocity of the particles from experiments. The velocity is normalized by the average velocity in the duct. The lines represent the channel fluid velocity, the linearized fluid velocity and the analytical fluid flow between 2 infinite plates.

In Figure S2 the fluid velocity profile normalized by the average fluid velocity in the duct in the middle of the channel, based on different approximations are depicted. The markers indicate the roughly the velocity of the particles along the z-direction when they are focused in the middle of the chip. Based on this analysis, it can be seen that the particle are situated between 0.4 and 0.6 μm, under the assumption that the particle is a point. Furthermore, it can be seen that the spread is very likely due to experimental error because there is no clear trend between the depth and the cross-flow velocity of the fluid. In addition, from this result, it can be observed that the depth is seemingly not governed by inertial lift forces between the wall and the particle, since there is no clear trend between fluid velocity and particle position.

However, a problem with the preceding analysis is that when the center of the particles is positioned at distances below 0.5 μm, the particle has to be partially inside the wall for the particles with a diameter of 1 μm, which is a non-physical result as a consequence of the point particle assumption. Therefore, this analysis is too simplistic and has to be modified. It is well known that a particle near a plane wall encounters more friction relative to a particle far from the wall and follows the fluid flow less well compared to particles far from the wall. This can be described by the lubrication theory as done in the past by for instance Goldman.[3] Pasol et al.[4] made an effort to transform all the lubrication friction factors into a Taylor series approximation, which can be used to calculate the velocity relative to the average velocity in the duct as a function of the distance of the particle from the wall. The velocity of the particles close to the wall is based on the velocity profile between 2 flat plates and is given by:[4]

$$u_p = 6u_0 \frac{y}{h} C_{lin} - \left((\frac{y}{h})^2 + \frac{\alpha^2}{3} \right) C_{qua} \tag{S5}$$

where C_{lin} is a correction term to the linear part of the particle velocity, C_{qua} is a correction term to the quadratic part of the velocity expression and α is the ratio of the particle radius over the channel height.

However, this result cannot be used directly, because the normalized velocity profile between 2 flat plates underestimates the flow profile in the middle of our chip (see Figure S2). Though, the velocity profile of the channel seems linear close to the wall. Therefore, by using another geometric correction factor of 1.3 to the linear part of the velocity profile for two flat plates, the velocity of the fluid in our chip can be approximated in the relevant distance from the wall (see Figure S2). Finally by including the geometric correction factor to the linear part of Equation S5 the gap between the particles and the electrode can be approximated more accurately.

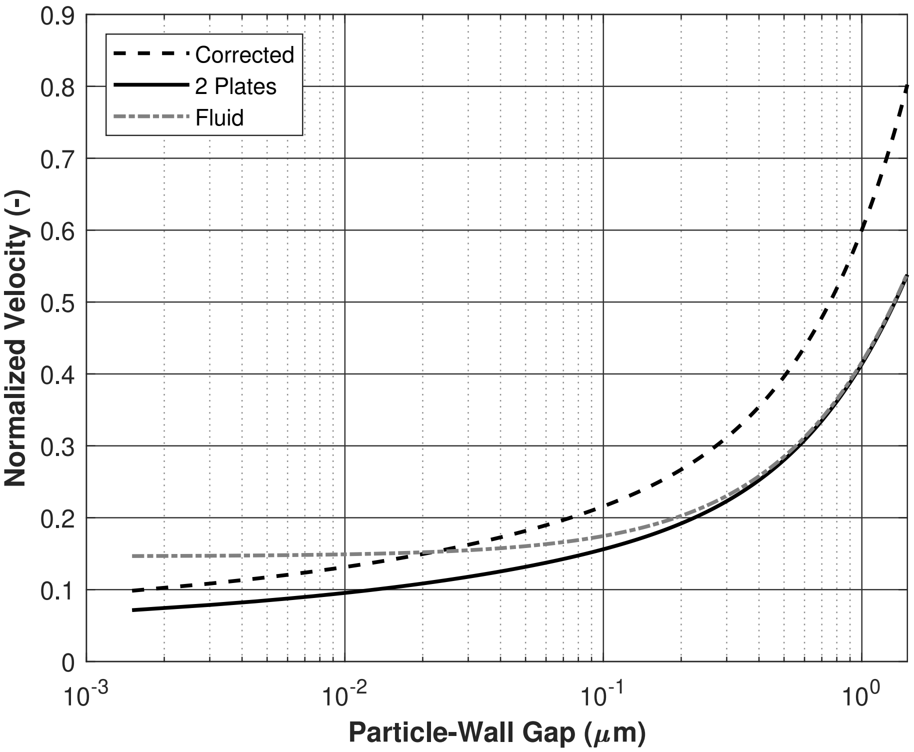

Figure S3: The fluid velocity relative to the average velocity in the channel versus the position along the height of the chip. For comparison, the 2 plate velocity profile and the linearized velocity profile in the channel are given.

In Figure S3, the velocity of the fluid flow between 2 plates, the velocity of the particles based on 2-plate velocity profile and the velocity of the particle according to the linearized channel velocity profile are depicted. From this figure, it is clear that at a distance of about 1 μm from the electrode wall, the particles follow the fluid velocity perfectly, though as the particle comes closer to the wall, the velocity of the particle drops more significantly compared to the fluid velocity due to the increased friction close to the wall is acting on the particles. Furthermore, due to the constant geometric correction factor, the velocity of the particles increases the two plate particle velocity by about 30 percent. From previous analysis, it was found that the experimentally determined normalized velocity of the particles was roughly between 0.1 and 0.2. According to the lubrication theory results this corresponds to a minimum gap between the particle and the wall of 10 nm, but it is more likely around a value of 80-50 nm. However, it should be noted that this is at most a rough

indication because the experimental velocity determination is a velocity composed of particles that are focused and particles that are about to get in the equilibrium position. Moreover, in the measured range, the change of particle velocity curve changes quite rapidly, which results in a broad range of possible particle gaps between the particle and the bottom electrode when the particles are focused.

S3 Force Balance with Different Particle Polarizability

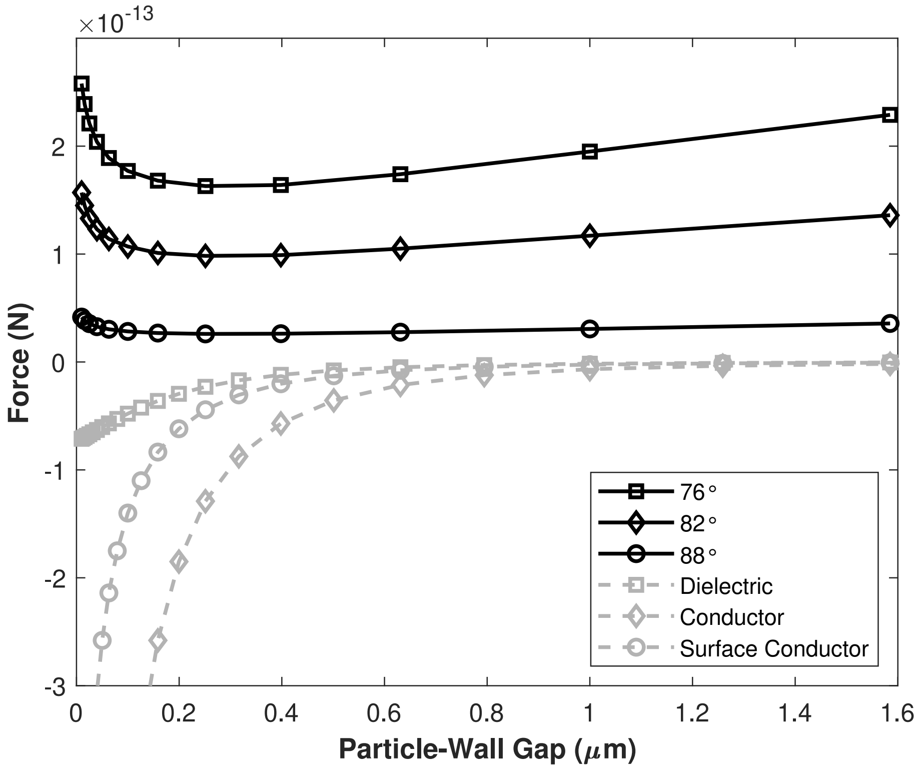

Figure S4: The hydrodynamic repulsive force for different acute angles of the trapezoidal geometry from simulations and the attractive induced dipole force in case of a dielectric, conductive and surface conductive particle.

The induced dipole force is related to the polarizability of the particle, which is determined by the dielectric constant or the conductivity of the particle. The 2 limits of this behavior

are a dielectric particle with a low dielectric constant relative to the medium and a particle with a very high dielectric constant that mimics a conductive particle, which corresponds to a Clausius-Mossotti factor of -0.5 and 1 respectively. In Figure S4 it can be seen that the case presented in the main text, which assumes that surface conduction of ions is occurring is in between the 2 limiting cases. Furthermore, these curves show that in principle it would be possible to selectively separate conductive particles from more dielectric particles in our chip. The geometry with an angle of minimally 82 °, would theoretically focus the conducting particles, while the dielectric particles would be repelled from the electrode by the repulsive hydrodynamic force.

S4 Hydrodynamic Force versus Stokes Force

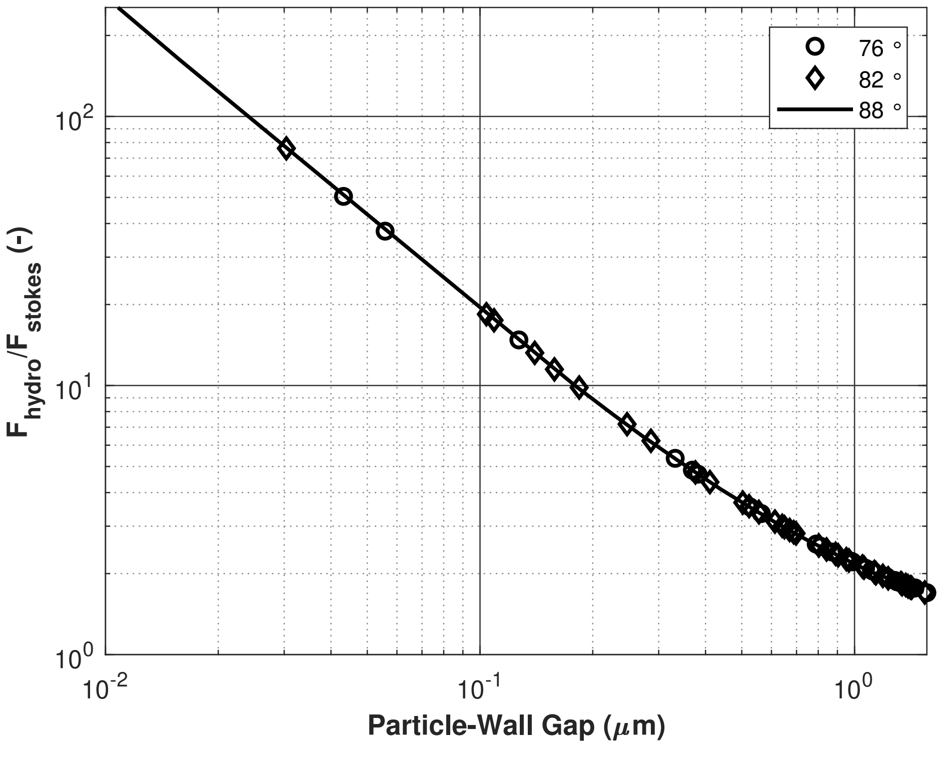

Figure S5: The ratio between the hydrodynamic force and the Stokes force force for geometries with different acute angles.

The hydrodynamic force on the particle is higher when the gap between the particle and the wall is relatively small and the difference decays exponentially with the gap size until it becomes unity far from the wall (see Figure S5). This shows that it is not sufficient to approximate the hydrodynamic repulsion by Stokes' law, since this will underestimate the force exerted on the particle considerably, especially at small gaps.

References

(1) Boussinesq, J. Mémoire sur l'influence des frottements dans les mouvements réguliers des fluids. *Journal de mathématiques pures et appliquées* **1868**, *13*, 377–424.

(2) Ruangkriengsin, T.; Roper, M. Approximating pressure-driven Stokes flow using the principle of minimal excess dissipation. *arXiv preprint arXiv:2204.07240* **2022**,

(3) Goldman, A.; Cox, R.; Brenner, H. Slow viscous motion of a sphere parallel to a plane wall—I Motion through a quiescent fluid. *Chem. Eng. Sci.* **1967**, *22*, 637–651.

(4) Pasol, L.; Martin, M.; Ekiel-Jezewska, M. L.; Wajnryb, E.; Bławzdziewicz, J.; Feuillebois, F. Motion of a sphere parallel to plane walls in a Poiseuille flow. Application to field-flow fractionation and hydrodynamic chromatography. *Chem. Eng. Sci.* **2011**, *66*, 4078–4089.
